# Additions to the Human Plasma Proteome via a Tandem MARS Depletion iTRAQ-Based Workflow

**DOI:** 10.1155/2013/654356

**Published:** 2013-02-19

**Authors:** Zhiyun Cao, Sachin Yende, John A. Kellum, Renã A. S. Robinson

**Affiliations:** ^1^Department of Chemistry, University of Pittsburgh, Pittsburgh, PA 15260, USA; ^2^The Clinical Research, Investigation, and Systems Modeling of Acute Illness (CRISMA) Laboratory Center and Department of Critical Care Medicine, University of Pittsburgh, Pittsburgh, PA 15260, USA

## Abstract

Robust platforms for determining differentially expressed proteins in biomarker and discovery studies using human plasma are of great interest. While increased depth in proteome coverage is desirable, it is associated with costs of experimental time due to necessary sample fractionation. We evaluated a robust quantitative proteomics workflow for its ability (1) to provide increased depth in plasma proteome coverage and (2) to give statistical insight useful for establishing differentially expressed plasma proteins. The workflow involves dual-stage immunodepletion on a multiple affinity removal system (MARS) column, iTRAQ tagging, offline strong-cation exchange chromatography, and liquid chromatography tandem mass spectrometry (LC-MS/MS). Independent workflow experiments were performed in triplicate on four plasma samples tagged with iTRAQ 4-plex reagents. After stringent criteria were applied to database searched results, 689 proteins with at least two spectral counts (SC) were identified. Depth in proteome coverage was assessed by comparison to the 2010 Human Plasma Proteome Reference Database in which our studies reveal 399 additional proteins which have not been previously reported. Additionally, we report on the technical variation of this quantitative workflow which ranges from ±11 to 30%.

## 1. Introduction

Discovery studies using plasma proteomics present challenges due to the technical difficulties associated with measuring the large dynamic range (~10–12 orders of magnitude) of proteins that exist in this medium [1]. Low-abundance proteins, which are of interest for biomarker applications, are often only accessible with involved proteomics workflows that utilize multiple sample fractionation steps. While the development of specific clinical immunoassays would resolve this approach, much work needs to be done in this area. Enrichment strategies for low-abundance plasma proteins rely on immunodepletion of high-abundance proteins [2–5], and, more recently, tandem depletion strategies have been employed [6–9]. For example, proteins present in as little as 1–1.6 *μ*g·mL^−1^ concentrations are detectable using tandem removal of abundant proteins with human serum albumin and Human 14 (Hu 14) multiple affinity removal system (MARS) columns [9]. A two-stage depletion setup that involves serial IgY and Supermix columns has also been effective in increasing the number of detectable low abundance proteins without affecting quantitative accuracy and precision using isobaric tags for relative and absolute quantification (iTRAQ) [6]. 

Recently, an updated reference database of human plasma proteins was released from the Human Proteome Organization which includes 1929 nonredundant protein sequences [10]. This list includes proteins that were identified amongst ~30 laboratories that utilized various enrichment and depletion strategies, shotgun proteomics techniques, and liquid, chromatography tandem mass spectrometry (LC-MS/MS) platforms. Herein, we report additions to the released reference database based on results obtained from the analysis of plasma samples in our laboratory analyzed by a dual depletion shotgun proteomics technique. 

Quantitative proteomics analyses of plasma are useful for identifying clinically relevant biomarkers [4] or in understanding disease mechanisms such as Alzheimer's disease [11]. The inherent biological variability across human patients can require a large number of samples in order to determine differentially expressed proteins that are statistically relevant. Depending on factors such as instrumental platform and available instrument time, multiplexing strategies are attractive. The commercial iTRAQ reagent allows up to eight samples to be multiplexed and has been effective in identifying biomarkers or differentially expressed proteins in diseases [12–14]. Limitations to this quantitative approach can include cost of reagent kits and issues with underestimation of ratios [15]. 

Nonetheless, iTRAQ can provide reliable quantitative information depending on the statistical rigor required for denoting proteins as differentially expressed [6, 16–19]. Several reports have stressed the importance of biological and technical replication in iTRAQ-based quantitative studies [13, 16, 19, 20]. These reports, however, do not converge on the same finite set of criteria for determining statistically relevant differentially expressed proteins. For example, Song et al. suggest that at least 20 or 8 biological samples are required in order to use fold change cutoff values of 1.5 and 2.0, respectively [19]. Chee et al. employ a ±30% or ±50% cutoff for technical and biological replicates, respectively [16]. Most recently, a fold change >2 was deemed appropriate when at least six biological sample replicates are employed in order to have sufficient statistical power [21]. That the criteria should even converge has also been questioned as it has been proposed that fold change cutoff values are dependent on many factors: replications, number of observed peptides, protein class (e.g., high or low abundance), and so forth; specific values should be defined based on experimental goals and design [22].

 Herein, we evaluate a robust tandem depletion quantitative proteomics workflow for its ability to provide additional insight to the human plasma proteome and to provide suitable criteria for the statistically relevant determination of differentially expressed proteins in human plasma. 

## 2. Materials and Methods

### 2.1. Plasma Samples

Four plasma samples were obtained from patients enrolled in the Genetic and Inflammatory Markers of Sepsis (GenIMS) study [23]. These patients were initially diagnosed with community-acquired pneumonia upon admittance to the emergency department, and samples were collected; however, further diagnoses revealed improper initial assessment. Thus these samples come from otherwise healthy volunteers. Approval for the participation of human subjects was obtained by the institutional review board of the University of Pittsburgh and other participating sites. 

### 2.2. Tandem MARS Immunodepletion (TMD)

The Hu 6 MARS column depletes serum albumin, IgG, *α*1-antitrypsin, IgA, transferrin, and haptoglobin proteins. An injection amount of 60 *μ*L of crude plasma was applied to the MARS column (Agilent; Santa Clara, CA, USA), and after the initial depletion, flow-through fractions were concentrated with a 5 K molecular weight cutoff concentrator (Agilent; Santa Clara, CA, USA) at 4695 g for 1.5 hours. Samples (hereafter referred to as MD) were then stored at −80°C or reinjected onto the MARS column for tandem MARS depletion. The second flow-through fractions (hereafter referred to as TMD) were concentrated, and protein concentrations were measured using the BCA protein assay. 

### 2.3. Protein Digestion and iTRAQ Labeling

In order to normalize experimental conditions, similar amounts of protein (i.e., 100 *μ*g) as determined from a BCA assay were employed. Protein amounts as opposed to sample volumes were used since the concentrations of proteins in the flow-through fraction may vary across samples after TMD. A total of 100 *μ*g of protein was denatured with an extraction buffer (0.2 M Tris, 8 M urea, 10 mM CaCl_2_, pH 8.0), reduced with 1 : 40 molar excess of dithiothreitol for 2 h at 37°C, and then alkylated with 1 : 80 molar excess of iodoacetamide for 2 h on ice in the dark. The alkylation reaction was quenched by adding 1 : 40 molar excess of cysteine, and the mixture was incubated at room temperature for 30 min. Molar excesses for each reagent was calculated based on an estimation of the total moles of protein in each sample (i.e., average MW of ~66 kDa). Tris buffer (0.2 M Tris, 10 mM CaCl_2_, pH 8.0) was added to dilute the urea concentration to 2 M. Each sample was incubated with bovine TPCK-heated trypsin at 50 : 1 substrate: enzyme mass ratio for 24 h at 37°C. Digested samples were desalted with an HLB cartridge (Waters; Milford, MA, USA) and dried by centrifugal evaporation. Each sample was labeled with an iTRAQ reagent following the manufacturer's protocol (Applied Biosystems; Foster City, CA, USA) with slight modifications. Briefly, each iTRAQ reagent was solubilized with 70 *μ*L ethanol and transferred to peptide mixtures. After 1.5 h of incubation, the reaction was quenched by adding 50 *μ*L of water. Labeled samples were mixed in 1 : 1 : 1 : 1 ratios for iTRAQ reagents that generate reporter ions *m/z* 114 : 115 : 116 : 117, respectively. 

### 2.4. Offline SCX Fractionation

For strong-cation exchange (SCX) liquid chromatography the separation was carried out on a polysulfoethyl 100 mm × 2.1 mm, 5 *μ*m, 200 Å column (The Nest Group Inc.; Southborough, MA, USA) with buffers as follows. Mobile phase A was 5 mM monopotassium phosphate (25% v/v acetonitrile, pH 3.0), and mobile phase B was 5 mM phosphate and 350 mM potassium chloride (25% v/v acetonitrile, pH 3.0). Dried iTRAQ-labeled samples were resuspended in 300 *μ*L of mobile phase A and injected onto the SCX column. The gradient for SCX was 0–3 min, 0% B; 3–45 min, 0–75% B; 45–50 min, 75–100% B; 50–55 min, 100% mobile phase B; 55–56 min, 100–0% B; and 56–106 min, 0% B. Thirteen SCX fractions were collected, and each fraction was desalted with an HLB cartridge. 

### 2.5. LC-MS/MS

Online desalting and reversed phase chromatography was performed with a Nano 2D-LC system equipped with an autosampler (Eksigent; Dublin, CA, USA). Mobile phase A and B for these analyses were 3% (v/v) acetonitrile with 0.1% formic acid and 100% (v/v) acetonitrile with 0.1% formic acid, respectively. SCX fractions were solubilized in 50 *μ*L of H_2_O with 0.1% formic acid and filtered with a 0.45 *μ*m filter (Thermo Fisher Scientific; Waltham, MA, USA). For each run, 5 *μ*L of sample was loaded into a trapping column (100 *μ*m i.d. × 2 cm), which was packed in-house with C_18_ 200 Å stationary phase material (Michrom Bioresource Inc.; Auburn, CA, USA) at 3 *μ*L·min^−1^ in 3% mobile phase B for 3 min. After desalting, the sample was loaded into the analytical column (75 *μ*m i.d.× 13.2 cm), which was packed in-house with C_18_ 100 Å stationary phase material (Michrom Bioresource Inc.). The gradient was as follows: 0–5 min, 10% mobile phase B; 5–75 min, 10–30% B; 75–95 min, 30–60% B; 95–100 min, 60–90% B; 100–105 min, 90–10% B; and 110–120 min, 10% B. The LC eluent was analyzed with positive-ion nanoflow electrospray using a LTQ-Orbitrap Velos mass spectrometer (Thermo-Fisher Scientific, Waltham, MA, USA). Data-dependent acquisition parameters were as follows: the MS survey scan in the Orbitrap was 60,000 resolution over 300–1800 *m/z*; CID was performed on the ion trap with normalized collision energy 35%; HCD was recorded in the Orbitrap with normalized collision energy 45% and 7,500 resolution; the top six most intense ions in the parent MS scan were selected and activated using CID and HCD [24]; dynamic exclusion was enabled with a repeat count of 2 for a duration of 60 sec; a minimum of 5000 ion counts were necessary for fragmentation events. Each fraction was subject to triplicate LC-MS/MS.

### 2.6. Database Searching

RAW files were analyzed with Proteome Discoverer 1.2 software (Thermo). Both CID and HCD spectra were used to obtain sequence information against the UniProt human database (04/25/2010, 20295 sequences). Sequest search parameters were as follows: enzyme specificity was trypsin with two maximum miscleavages; precursor mass tolerance was 10 ppm; fragment mass tolerance was 0.8 Da; N-terminus and lysine modification with iTRAQ (144.102 Da) and cysteine carbamidomethylation (57.021 Da) were set as fixed modifications; tyrosine modification with iTRAQ was set as a dynamic modification. Decoy database searching was employed to generate medium (*P* < 0.05) and high (*P* < 0.01) confidence peptide lists. All peptides with medium and high confidence were pooled into a single data file and used for final protein identification and quantitation. Proteins with at least two spectral counts in a workflow replicate were included for identification. Only proteins with at least two spectral counts in a technical replicate were considered for quantitative and statistical analysis. One-way ANOVA analysis (*P* < 0.05) was performed for proteins quantified in at least two workflow replicates utilizing Microsoft Excel.

### 2.7. Protein Quantification and Statistical Analysis

Peptide ratios (e.g., 115/114, 116/114, and 117/114) were calculated based on the peak intensity of each reporter ion. The protein ratios were the median ratio of the corresponding peptide ratios. Coefficients of variation (CV) values were calculated for ratios of proteins quantified in at least two workflow replicates. The mean CV value across workflow replicates was calculated and used as the total biological variation, *S*_*b*_. The technical variation, *S*_*t*_, was calculated for proteins quantified in at least two LC-MS/MS analyses within an individual workflow. The relation between the fold change (*F*), random variation (*S*), biological replicates per group (*n*), and technical replicates (*m*) has been previously reported [25] and is expressed by the formula

(1) $$\begin{array}{c} n=\frac{\;2{\left(Z+T\right)}^{2}S^{2}}{{\left(F-1\right)}^{2}\;\;},\\ S={\left(\frac{S_{b}^{2}\;+\;S_{t}^{2}}{m}\right)}^{1/2}. \end{array}$$

The quantities *Z* and *T* depend on the power of the test and the significance level, respectively. The power and significance levels were set as 0.8 and 0.05, respectively, such that the formula approximates to

(2) $$\begin{array}{c} n=\frac{20S^{2}}{{\left(F-1\right)}^{2}},\\ F=\frac{4.47S}{n^{1/2}}+1. \end{array}$$

One-way ANOVA analysis (*P* < 0.05) was performed for proteins quantified in at least two workflow replicates utilizing Microsoft Excel.

## 3. Results and Discussion

**Figure 1:**
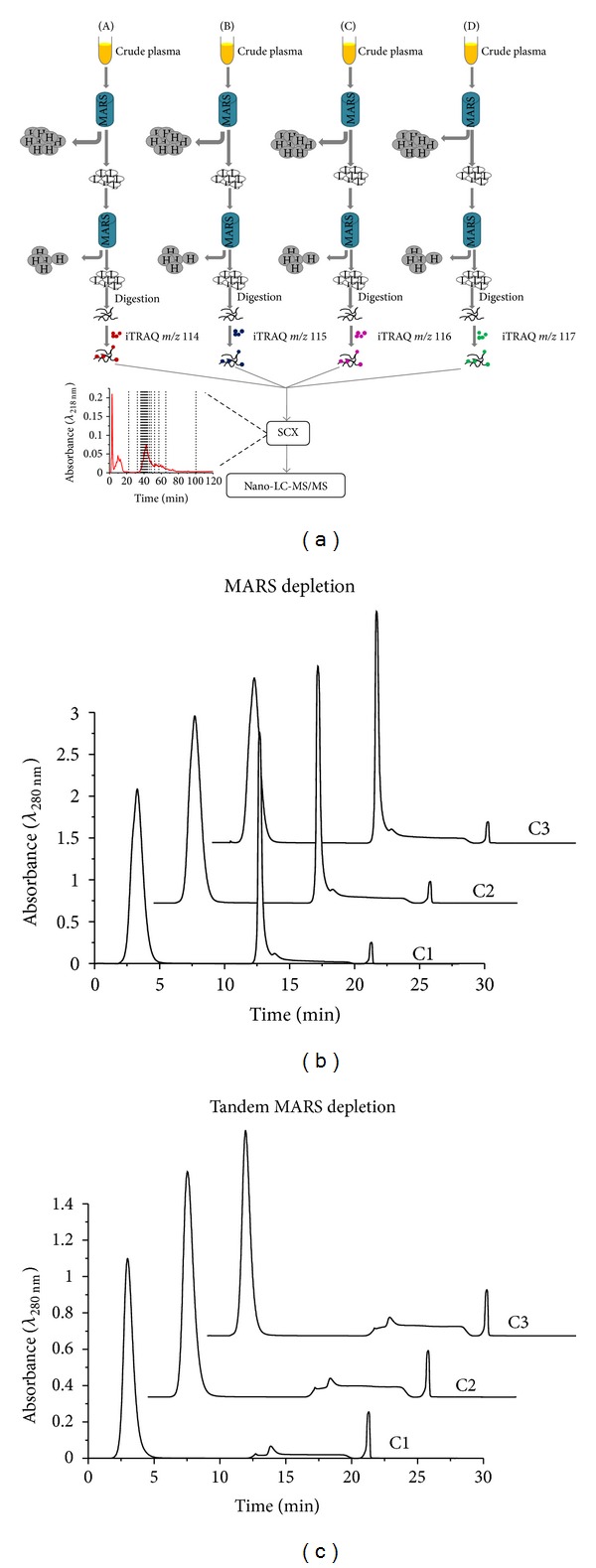
(a) The iTRAQ-based quantitative platform used for plasma proteome analyses in which the flow-through fractions from four crude plasma samples (A–D) are modified with iTRAQ 4-plex reagents, pooled into a single mixture, and separated with offline SCX-LC-MS/MS. Example chromatograms (*λ*_280 nm_) from three independent injections of plasma sample C upon (b) MD and (c) TMD are shown.

A robust quantitative shotgun proteomics workflow (Figure 1(a)) was assessed for its ability to identify new human plasma proteins and to guide future experimental designs. The workflow uses tandem MARS depletion (TMD), iTRAQ four-plex reagents, SCX fractionation, and nanoflow LC-MS/MS on a LTQ-Orbitrap Velos MS. The entire workflow was repeated three times using new aliquots of four plasma samples that were subject to TMD using a Hu 6 MARS column. The time it takes to complete a single workflow replicate is ~7 days with a majority of the costs being attributed to the MARS column (~200 analyses per column) and the iTRAQ reagents (5 analyses per kit). Immunodepletion of samples is very reproducible for single-stage MARS depletion (MD, Figure 1(b)) and TMD (Figure 1(c)). It is apparent from the chromatograms (Figures 1(b) and 1(c)) that high abundance proteins (i.e., *t*_*r*_ ~ 12.5 min) are substantially depleted after the TMD step. The average % depletion of the six high abundance proteins is 88% and 92% for MD and TMD, respectively, (see Supplementary Table S1 of the Supplementary Material available online at http://dx.doi.org/10.1155/2013/654356) and is similar to that obtained using other tandem depletion strategies [6, 8, 9]. It should be noted that albumin was still detectable after TMD (Supplementary Table S2); however other abundant proteins (i.e., *α*-1-antitrypsin, IgG, IgA, transferrin, and haptoglobin) did not have any observed peptide hits. The most abundant protein detected based on spectral counts was complement C3 which had an average total spectral count (SC) of >4000 across the workflow replicates (Supplementary Table S2). The use of a single column to perform dual immunodepletion minimizes the expenses associated with the use of multiple MARS or other depletion columns. 

**Figure 2:**
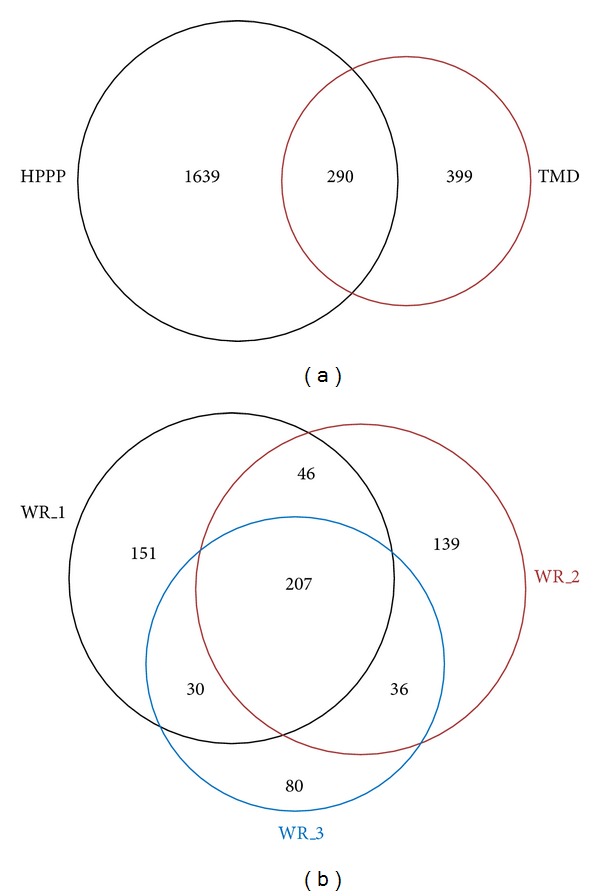
(a) Venn diagram for proteins identified in the human plasma proteome project (HPPP) and the TMD workflow presented herein. (b) Venn diagram for proteins identified in three workflow replicate (WR) experiments.

TMD samples were used for further iTRAQ tagging reactions and analyzed with SCX LC-MS/MS (Figure 1(a)). A total of 689 unique proteins were identified from the combined results of the three independent workflow experiments (Supplementary Table S2) and are slightly larger than the number of proteins observed in other reports [5, 9, 19, 26, 27]. The proteins identified in this study were compared to the recently released 2011 HUPO plasma protein database to assess the depth of proteome coverage. Based on comparisons of identified proteins to the 1929 nonredundant sequences reported in the Human Proteome Organization Database [10], 399 novel proteins with ≥2 SC are uniquely observed in these studies (Figure 2(a)). Although the incorporation of a dual depletion step and SCX fractionation increases experimental sample preparation time, our results support the necessity of these (or similar) steps for identification of commonly detected and novel plasma proteins. Due to different experimental designs, LC-MS/MS data acquisition settings, and searching engines, the number of identified proteins may vary a lot across different labs. It is also possible that a portion of the identifications are a result of profiles specific to the patient samples employed. All of the proteins identified are provided in Supplementary Table S2. A total of 207 proteins were observed in all three of the workflow experiments, and more than half of the total proteins were observed in a single workflow replicate (Figure 2(b)). With more stringent criteria (i.e., not less than 2 unique peptides for protein identification), 229 proteins were identified across three workflow replicates, and 40 new proteins were identified in these studies in comparison to the HUPO database. 

The datasets collected from this TMD strategy were used to examine the variation in the entire workflow. iTRAQ reporter ion (i.e., *m/z* 115, 116, and 117) ratios were calculated with respect to *m/z* 114 for each protein. Proteins quantified by at least 2 spectral counts were used in the assessment of variation. Of the 207 proteins identified in all three workflow replicates, 139 proteins (with at least 2 spectral counts) were quantified in the Proteome Discoverer Analysis. These proteins were used to initially assess the variance in reporter ion ratios across the workflow replicates (of which each includes three technical replicates) by employing well-established statistical approaches [25, 28–31]. We refer to a technical replicate as the cumulative results obtained across individual LC-MS/MS analyses of the 13 SCX fractions. Thus within a single workflow experiment three technical replicates were measured. The workflow replicate assesses the variation beginning with the start of the plasma sample preparation. 

**Figure 3:**
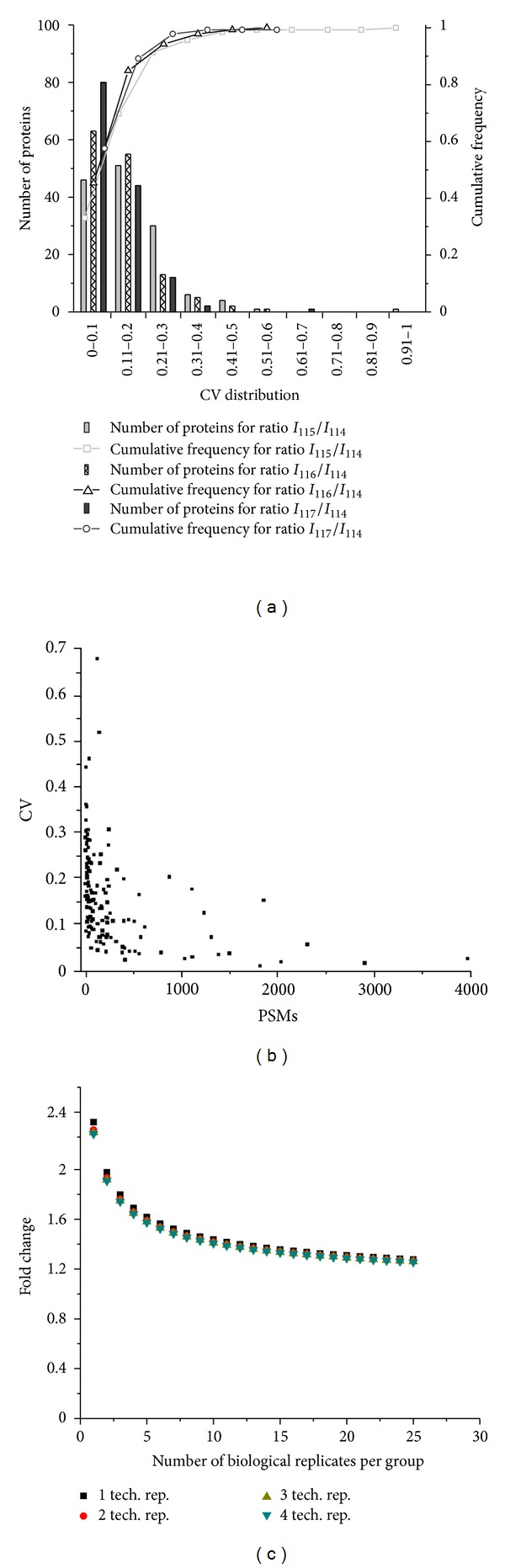
(a) The distribution of CV values for *I*_115_/*I*_114_ (grey rectangular), *I*_116_/*I*_114_ (shaded rectangular), and *I*_117_/*I*_114_ (black rectangular) for proteins quantified in each of the three independent experiments (*N* = 139 proteins). The cumulative frequency of proteins with specific CV values for *I*_115_/*I*_114_ (dashed square), *I*_116_/*I*_114_ (dashed triangle), and *I*_117_/*I*_114_ (dashed circle) are shown. CV values are given as fractional values. The total peak intensity is represented by *I*. (b) Plot of the mean CV values for reporter ion ratios relative to reference channel 114 as a function of the number of spectral counts identified for each protein. Only proteins identified in all three workflow replicates are represented in this plot. (c) Power analysis for iTRAQ-based quantitative platform whereby fold change values are plotted for a given number of biological replicates as a function of the number of technical replicates (i.e., *m* = 1 to 4). The power and significance level values were set to 80% and 0.05, respectively.

Figure 3(a) plots the distribution of CV values for proteins as a function of reporter ion ratios (e.g., 115/114, 116/114, and 117/114). The distribution of SD values for proteins as a function of log_2_ transformed ratios are provided in Supplementary Figure 1. Within a single workflow replicate, the average reporter ion ratio across technical replicates was calculated for individual proteins. The corresponding mean (and median) CV values for ratios 115/114, 116/114, and 117/114 across all proteins quantified in the three workflow replicates was ±0.16 (0.13), 0.13 (0.11), and 0.11 (0.09), respectively. Seventy-five percent of proteins had a CV <0.16, and 90% of proteins had a CV <0.21 when reporter ion 114 was used as the reference channel. Because the reporter ion channel used as the reference can have some effect on quantitation [19], the mean (and median) CV values were also calculated for different reference channels (Supplementary Table S3). When reporter ions *m/z* 115, 116, and 117 were used as the reference channel, 90% of proteins had a CV value <0.28, 0.21, and 0.24, respectively. This range of CV values that results from selection of different reference channels reflects the variation inherent in the four plasma samples as well as any variation that arises during LC-MS/MS analysis. 

Incorporation of multiple workflow or technical replicates does not imply that proteins will be observed in all experiments (Figure 2(a)); therefore CV values were also calculated for the 71 proteins that were only quantified in any two of the three workflow replicates. When reporter ion *m/z* 114 was used as the reference channel, the mean (and median) CV was ±0.30 (0.23), 0.20 (0.15), and 0.18 (0.15) for ratios 115/114, 116/114, and 117/114, respectively (Supplementary Table S3). The higher CV observed for this set of proteins agrees with the notion that less replication (workflow and technical) could lead to higher variation in reporter ion ratios [20, 28] as well as biases that arise in low abundance proteins due to lower numbers of detected spectral counts and higher variability due to lower intensity signals [22]. Higher variability in reporter ion ratios correlated with proteins that were identified with lower numbers of spectral counts (Figure 3(b)). 

In order to estimate the overall variance of this workflow, CV values were obtained for proteins quantified in at least two of the workflow replicates (*N* = 210). The mean CV was 0.21, 0.15, and 0.13 for ratios 115/114, 116/114, and 117/114, respectively, and similar values were obtained for other reference channels (Supplementary Table S3). Taking the CV values of reference channel 114 into consideration, the overall variation in the entire plasma workflow is ~0.16. Herein, the technical variation was assessed by considering proteins observed in multiple LC-MS/MS analyses for individual workflow replicates. The technical variation is ~0.10 for proteins quantified in at least two replicates (Supplementary Table S4). In order to determine proteins that were quantified similarly across workflow replicates, one-way ANOVA analysis (*P* < 0.05) [31] was carried out. Based on these results, ~70% of the 210 quantified proteins have similar ratios across workflow replicates (*data not shown*). 

Power analysis was also performed in order to assess the fold-change criterion that should be applied based on a given number of biological replicates (Figure 3(c)). We note that our experimental approach (i.e., repeating the workflow using new aliquots of the same plasma samples) does not represent a true biological replicate. However, this analysis still provides statistical insight to the power of biological replication in future experimental designs. The total biological variance (*S*_*b*_), technical variance (*S*_*t*_), power, and significance level applied were 0.16, 0.10, 80%, and 0.05, respectively. As indicated in Figure 3(c), if ten biological replicates per group are used then a fold-change cutoff of 1.3 can be applied, and only two replicates are required to use the commonly applied 2.0 fold-change cutoff. Technical replicates do not appear to have a significant effect on the fold-change criterion when multiple biological replicates will be used (Figure 3(c)). These data provide additional evidence to support the notion that biological replication (i.e., in these studies workflow replication) is one of the most important factors that should be considered in the experimental design [16, 21, 25]. 

This paper has presented a robust quantitative plasma proteomics workflow that involves tandem MARS depletion, iTRAQ tagging, and SCX-LC-MS/MS analysis. The use of TMD and SCX fractionation resulted in the identification of 689 proteins with ≥2 SC. Compared to the HUPO database, ~400 of these proteins were previously unreported. The use of TMD and SCX fractionation significantly increases the number of proteins detected. The overall variation in the presented workflow ranges from ±11 to 30%, and power analysis indicates that increasing biological replication would allow a lower fold-change cutoff to be applied to determine statistically relevant differentially expressed proteins. Future studies from our laboratory involve the application of this workflow to specific disease states whereby biological replicates are also being incorporated into the experimental design.

## Supplementary Material

“The Supplementary Material provides the following information: Table S1) Depletion efficiency of the six high abundance proteins with MD and TMD; Table S2) A list of proteins identified with corresponding spectral counts in each workflow replicate; Table S3) CV values for proteins quantified in workflow replicates; Table S4) CV values for proteins quantified in at least two technical replicates, and Figure S1) The distribution of SD values for proteins quantified in all workflow replicates as a function of log2 transformed ratios.”
