# Supplementary material for: Additions to the Human Plasma Proteome via a Tandem MARS Depletion iTRAQ-Based Workflow
Source: Int J Proteomics. 2013 Feb 19;2013:654356. doi: 10.1155/2013/654356 (PMC3590782; doi:10.1155/2013/654356)
Supplement: Supplementary file 1 — “The Supplementary Material provides the following information: Table S1) Depletion efficiency of the six high abundance proteins with MD and TMD; Table S2) A list of proteins identified with corresponding spectral counts in each workflow replicate; Table S3) CV values for proteins quantified in workflow replicates; Table S4) CV values for proteins quantified in at least two technical replicates, and Figure S1) The distribution of SD values for proteins quantified in all workflow replicates as a function of log2 transformed ratios.” [file 654356.f1.zip › TableS1.pdf]

Table 1. Comparison of % depletion across the plasma samples tested.

Plasma Sample	% MD[a]	Mean ± SD (%)	% TMD[b]	Mean ± SD (%)
**A1**	89.1		92.9	
**A2**	87.6		91.8	
**A3**	83.5	86.7 ± 2.9	89.2	91.3 ± 1.9
**B1**	88.1		91.7	
**B2**	85.9		90.8	
**B3**	87.1	87.0 ± 1.1	91.7	91.4 ± 0.5
**C1**	88.9		94.0	
**C2**	86.7		91.2	
**C3**	88.6	88.1 ± 1.2	91.9	92.4 ± 1.5
**D1**	90.1		95.2	
**D2**	91.1		94.5	
**D3**	87.2	89.5 ± 2.0	91.7	93.8 ± 1.9

[a]% MARS depletion (MD), [b]%tandem MARS depletion (TMD). Values calculated based on protein amounts, determined from BCA
