# Supplementary material for: Additions to the Human Plasma Proteome via a Tandem MARS Depletion iTRAQ-Based Workflow
Source: Int J Proteomics. 2013 Feb 19;2013:654356. doi: 10.1155/2013/654356 (PMC3590782; doi:10.1155/2013/654356)
Supplement: Supplementary file 1 — “The Supplementary Material provides the following information: Table S1) Depletion efficiency of the six high abundance proteins with MD and TMD; Table S2) A list of proteins identified with corresponding spectral counts in each workflow replicate; Table S3) CV values for proteins quantified in workflow replicates; Table S4) CV values for proteins quantified in at least two technical replicates, and Figure S1) The distribution of SD values for proteins quantified in all workflow replicates as a function of log2 transformed ratios.” [file 654356.f1.zip › TableS2.pdf]

Table S2. List of proteins identified in each workflow replicate.

Accession No.[a]	Protein Name	MW[b] (kDa)	SCs[c]		
			WR1[d]	WR2[d]	WR3[d]
A0AVT1	Ubiquitin-like modifier-activating enzyme 6	117.90		2	
A2BFH1	Peptidylprolyl cis-trans isomerase A-like 4A	18.15		3	
A4D2C0	Postmeiotic segregation increased 4-like protein	27.96			2
A5A3E0	POTE ankyrin domain family member F	121.37	61	14	12
A5PLK6	Regulator of G-protein signaling protein-like	125.61	2	2	
A6NC57	Ankyrin repeat domain-containing protein 62	104.75	2		
A6NHZ5	Leucine-rich repeat-containing protein 14-like	56.37	2		
A8MVM7	Putative uncharacterized protein ENSP00000382790	73.41		8	3
A8MW06	Thymosin beta-4-like protein 3	5.06		2	
A8MYB1	Transmembrane and coiled-coil domain-containing protein 5B	35.74	6	6	6
B1AJZ9	Forkhead-associated domain-containing protein 1	161.80		2	
O00187	Mannan-binding lectin serine protease 2	75.68	10	6	3
O00214	Galectin-8	35.79			2
O00391	Sulfhydryl oxidase 1	82.53	14	17	17
O00533	Neural cell adhesion molecule L1-like protein	134.97	16	7	
O00567	Nucleolar protein 56	66.01	2		
O00743	Serine/threonine-protein phosphatase 6 catalytic subunit	35.12		6	
O14495	Lipid phosphate phosphohydrolase 3	35.09	2		
O14686	Histone-lysine N-methyltransferase MLL2	563.83	2		
O14744	Protein arginine N-methyltransferase 5	72.64	3		
O14753	Putative transcription factor Ovo-like 1	30.24	5		
O14786	Neuropilin-1	103.07	7	3	
O14791	Apolipoprotein L1	43.95	50	40	36
O14929	Histone acetyltransferase type B catalytic subunit	49.48		2	
O15230	Laminin subunit alpha-5	399.54	4	3	2
O43143	Putative pre-mRNA-splicing factor ATP-dependent RNA helicase DHX15	90.88	2	4	
O43299	Uncharacterized protein KIAA0415	88.55	2	3	4
O43423	Acidic leucine-rich nuclear phosphoprotein 32 family member C	26.75	2		
O43439	Protein CBFA2T2	67.09		2	
O43490	Prominin-1	97.14			2
O43866	CD5 antigen-like	38.06	8	6	
O60287	Nucleolar pre-ribosomal-associated protein 1	254.23	3		
O60496	Docking protein 2	45.35			2
O60825	6-phosphofructo-2-kinase/fructose-2,6-biphosphatase 2	58.44	6		
O60879	Protein diaphanous homolog 2	125.49		3	
O60885	Bromodomain-containing protein 4	152.12		3	2
O75015	Low affinity immunoglobulin gamma Fc region receptor III-B	26.20	8		
O75037	Kinesin-like protein KIF21B	182.55		4	5
O75127	Pentatricopeptide repeat-containing protein 1	78.81			2

O75128	Protein cordon-bleu	135.53	4		
O75369	Filamin-B	277.99			2
O75496	Geminin	23.55		3	
O75525	KH domain-containing, RNA-binding, signal transduction-associated protein 3	38.78	6		
O75636	Ficolin-3	32.88	28	62	31
O75882	Attractin	158.43	37	27	44
O76031	ATP-dependent Clp protease ATP-binding subunit clpX-like, mitochondrial	69.18		6	
O95025	Semaphorin-3D	89.59		3	3
O95361	Tripartite motif-containing protein 16	63.91	2		
O95445	Apolipoprotein M	21.24	14	18	15
O95567	Uncharacterized protein C22orf31	32.63		2	
O95685	Protein phosphatase 1 regulatory subunit 3D	32.54			2
O95789	Zinc finger MYM-type protein 6	147.99	8		
O95996	Adenomatous polyposis coli protein 2	243.80	3		
P00338	L-lactate dehydrogenase A chain	36.67	2		
P00450	Ceruloplasmin	122.13	1455	1462	1042
P00488	Coagulation factor XIII A chain	83.21	43	24	20
P00734	Prothrombin	69.99	519	472	396
P00736	Complement C1r subcomponent	80.07	288	151	193
P00740	Coagulation factor IX	51.74	56	34	26
P00742	Coagulation factor X	54.70	32	49	31
P00746	Complement factor D	27.02	7	10	7
P00747	Plasminogen	90.51	626	446	357
P00748	Coagulation factor XII	67.77	53	61	54
P00751	Complement factor B	85.48	1078	1086	954
P00915	Carbonic anhydrase 1	28.85	27	20	23
P00918	Carbonic anhydrase 2	29.23	4	2	3
P00995	Pancreatic secretory trypsin inhibitor	8.50			4
P01008	Antithrombin-III	52.57	694	509	368
P01011	Alpha-1-antichymotrypsin	47.62	1891	1217	1071
P01019	Angiotensinogen	53.12	246	233	186
P01023	Alpha-2-macroglobulin	163.19	3694	2798	2275
P01024	Complement C3	187.03	4536	4126	3341
P01031	Complement C5	188.19	424	420	336
P01034	Cystatin-C	15.79	22	30	42
P01042	Kininogen-1	71.91	933	788	660
P01343	Insulin-like growth factor IA	17.01	3	9	
P01344	Insulin-like growth factor II	20.13	9		6
P01857	Ig gamma-1 chain C region	36.08	9	6	5
P01859	Ig gamma-2 chain C region	35.88	3	2	
P01860	Ig gamma-3 chain C region	41.26	7	3	
P02042	Hemoglobin subunit delta	16.05	4		2
P02533	Keratin, type I cytoskeletal 14	51.53	43		12
P02538	Keratin, type II cytoskeletal 6A	60.01	45		29
P02647	Apolipoprotein A-I	30.76	3032	2153	1773

P02649	Apolipoprotein E	36.13	219	228	216
P02652	Apolipoprotein A-II	11.17	687	440	430
P02654	Apolipoprotein C-I	9.33	121	74	95
P02655	Apolipoprotein C-II	11.28	69	53	58
P02656	Apolipoprotein C-III	10.85	76	85	53
P02671	Fibrinogen alpha chain	94.91	1151	1186	1014
P02675	Fibrinogen beta chain	55.89	2008	1914	1685
P02679	Fibrinogen gamma chain	51.48	1468	1229	1031
P02735	Serum amyloid A protein	13.52	783	490	419
P02741	C-reactive protein	25.02	60	72	70
P02743	Serum amyloid P-component	25.37	50	38	37
P02745	Complement C1q subcomponent subunit A	26.00	44	26	29
P02746	Complement C1q subcomponent subunit B	26.44	51	36	33
P02747	Complement C1q subcomponent subunit C	25.76	50	64	32
P02748	Complement component C9	63.13	171	186	188
P02749	Beta-2-glycoprotein 1	38.27	347	478	372
P02750	Leucine-rich alpha-2-glycoprotein	38.15	276	254	168
P02751	Fibronectin	262.44	242	191	158
P02753	Retinol-binding protein 4	23.00	127	76	78
P02760	Protein AMBP	38.97	271	189	189
P02763	Alpha-1-acid glycoprotein 1	23.50	1140	871	643
P02765	Alpha-2-HS-glycoprotein	39.30	323	231	201
P02766	Transthyretin	15.88	274	134	63
P02768	Serum albumin	69.32	215	93	65
P02774	Vitamin D-binding protein	52.93	619	507	406
P02775	Platelet basic protein	13.89	22	19	22
P02776	Platelet factor 4	10.84	10	10	4
P02787	Serotransferrin	77.00	3		
P02788	Lactotransferrin	78.13	2		
P02790	Hemopexin	51.64	2036	1355	1128
P03951	Coagulation factor XI	70.06	6		
P03952	Plasma kallikrein	71.32	178	123	148
P04003	C4b-binding protein alpha chain	66.99	43	18	19
P04004	Vitronectin	54.27	320	216	212
P04040	Catalase	59.72	2	6	2
P04070	Vitamin K-dependent protein C	52.04	16	2	2
P04114	Apolipoprotein B-100	515.24	2231	2049	1857
P04156	Major prion protein	27.64	2		
P04180	Phosphatidylcholine-sterol acyltransferase	49.55	41	17	16
P04196	Histidine-rich glycoprotein	59.54	259	233	206
P04217	Alpha-1B-glycoprotein	54.24	640	585	470
P04259	Keratin, type II cytoskeletal 6B	60.03	77		
P04264	Keratin, type II cytoskeletal 1	66.00	459	291	254
P04275	von Willebrand factor	309.06	42	60	60
P04278	Sex hormone-binding globulin	43.75	14	7	5
P05090	Apolipoprotein D	21.26	85	85	70
P05154	Plasma serine protease inhibitor	45.67	41	11	17

P05155	Plasma protease C1 inhibitor	55.12	419	454	338
P05156	Complement factor I	65.68	190	146	124
P05160	Coagulation factor XIII B chain	75.46	79	58	45
P05362	Intercellular adhesion molecule 1	57.79	17	2	8
P05451	Lithostathine-1-alpha	18.72		5	2
P05452	Tetranectin	22.55	108	98	68
P05543	Thyroxine-binding globulin	46.29	100	64	64
P05546	Heparin cofactor 2	57.03	241	244	216
P05556	Integrin beta-1	88.36	2		
P06276	Cholinesterase	68.37	33	32	35
P06396	Gelsolin	85.64	253	297	254
P06681	Complement C2	83.21	183	179	122
P06702	Protein S100-A9	13.23	6		2
P06727	Apolipoprotein A-IV	45.37	389	426	351
P07225	Vitamin K-dependent protein S	75.07	31	50	47
P07333	Macrophage colony-stimulating factor 1 receptor	107.92	6	4	2
P07357	Complement component C8 alpha chain	65.12	147	126	106
P07358	Complement component C8 beta chain	67.00	183	119	95
P07359	Platelet glycoprotein Ib alpha chain	68.91	14	4	2
P07360	Complement component C8 gamma chain	22.26	161	124	113
P07437	Tubulin beta chain	49.64		2	
P07477	Trypsin-1	26.54	18	40	41
P07478	Trypsin-2	26.47	5	2	6
P07585	Decorin	39.72	4		
P07602	Proactivator polypeptide	58.07	4	3	
P07737	Profilin-1	15.04	8		
P07996	Thrombospondin-1	129.30	12		3
P07998	Ribonuclease pancreatic	17.63	6	6	4
P08174	Complement decay-accelerating factor	41.37	8	7	13
P08185	Corticosteroid-binding globulin	45.11	12	6	10
P08294	Extracellular superoxide dismutase [Cu-Zn]	25.83	5	6	
P08519	Apolipoprotein(a)	501.00	7		
P08571	Monocyte differentiation antigen CD14	40.05	26	14	11
P08603	Complement factor H	139.00	1247	1096	1016
P08637	Low affinity immunoglobulin gamma Fc region receptor III-A	29.07			5
P08697	Alpha-2-antiplasmin	54.53	133	228	182
P08709	Coagulation factor VII	51.56	8	4	
P08779	Keratin, type I cytoskeletal 16	51.24	43		
P09172	Dopamine beta-hydroxylase	69.02	6	2	3
P09327	Villin-1	92.64	2		
P09630	Homeobox protein Hox-C6	26.90			2
P09758	Tumor-associated calcium signal transducer 2	35.69			2
P09871	Complement C1s subcomponent	76.63	213	223	167
P0C091	FRAS1-related extracellular matrix protein 3	237.62		2	
P0C0L4	Complement C4-A	192.65	1953	1181	
P0C0L5	Complement C4-B	192.67	1978	1833	1678

P10643	Complement component C7	93.46	281	268	227
P10909	Clusterin	52.46	263	234	179
P11021	78 kDa glucose-regulated protein	72.29		5	
P11137	Microtubule-associated protein 2	199.40	3		
P11142	Heat shock cognate 71 kDa protein	70.85	4	3	2
P11234	Ras-related protein Ral-B	23.39			3
P11532	Dystrophin	426.43	4	10	2
P12111	Collagen alpha-3(VI) chain	343.45	2		
P12259	Coagulation factor V	251.51	40	22	31
P12814	Alpha-actinin-1	102.99	5	5	3
P12830	Cadherin-1	97.40	2		
P12883	Myosin-7	222.96		12	5
P12931	Proto-oncogene tyrosine-protein kinase Src	59.80		2	
P12955	Xaa-Pro dipeptidase	54.51	2		
P13598	Intercellular adhesion molecule 2	30.63	4	6	
P13611	Versican core protein	372.59		3	
P13645	Keratin, type I cytoskeletal 10	58.79	156	136	151
P13647	Keratin, type II cytoskeletal 5	62.34	59	20	22
P13671	Complement component C6	104.72	193	194	162
P13796	Plastin-2	70.24	4		2
P13805	Troponin T, slow skeletal muscle	32.93		2	
P14151	L-selectin	42.16	22	13	17
P14618	Pyruvate kinase isozymes M1/M2	57.90	3		
P14927	Cytochrome b-c1 complex subunit 7	13.52	2		
P15144	Aminopeptidase N	109.47	2		
P15151	Poliovirus receptor	45.27	3	12	
P15169	Carboxypeptidase N catalytic chain	52.25	59	73	68
P15259	Phosphoglycerate mutase 2	28.75	6		
P16070	CD44 antigen	81.50	24	22	27
P16278	Beta-galactosidase	76.03	4		
P17019	Zinc finger protein 708	64.76	5	3	4
P17023	Zinc finger protein 19	52.42	2		2
P17480	Nucleolar transcription factor 1	89.35			3
P17858	6-phosphofructokinase, liver type	84.96			2
P17936	Insulin-like growth factor-binding protein 3	31.65	37	26	27
P18065	Insulin-like growth factor-binding protein 2	34.79		6	
P18074	TFIIH basal transcription factor complex helicase subunit	86.85		2	
P18206	Vinculin	123.72	4		2
P18428	Lipopolysaccharide-binding protein	53.35	69	46	34
P19320	Vascular cell adhesion protein 1	81.22	9		7
P19652	Alpha-1-acid glycoprotein 2	23.59	237	275	230
P19823	Inter-alpha-trypsin inhibitor heavy chain H2	106.40	455	386	396
P19827	Inter-alpha-trypsin inhibitor heavy chain H1	101.33	411	302	257
P20061	Transcobalamin-1	48.18	2		2
P20273	B-cell receptor CD22	95.29			2
P20700	Lamin-B1	66.37		5	2

P20701	Integrin alpha-L	128.69			2
P20742	Pregnancy zone protein	163.76	457	203	217
P20851	C4b-binding protein beta chain	28.34	2	3	3
P21266	Glutathione S-transferase Mu 3	26.54	4	4	5
P21333	Filamin-A	280.56	22	7	3
P21817	Ryanodine receptor 1	564.82	2		2
P22105	Tenascin-X	464.03	7		2
P22352	Glutathione peroxidase 3	25.54	37	63	33
P22692	Insulin-like growth factor-binding protein 4	27.92	2		2
P22792	Carboxypeptidase N subunit 2	60.58	133	118	107
P22891	Vitamin K-dependent protein Z	44.71	8	7	8
P22897	Macrophage mannose receptor 1	165.90	7		2
P23141	Liver carboxylesterase 1	62.48		2	
P23142	Fibulin-1	77.16	18	10	13
P23528	Cofilin-1	18.49	4		
P23919	Thymidylate kinase	23.80	2		
P24043	Laminin subunit alpha-2	343.68	13	3	6
P24723	Protein kinase C eta type	77.78	3		
P25021	Histamine H2 receptor	40.07		2	2
P25054	Adenomatous polyposis coli protein	311.45			2
P25116	Proteinase-activated receptor 1	47.41		4	2
P25311	Zinc-alpha-2-glycoprotein	34.24	465	398	341
P26373	60S ribosomal protein L13	24.25		6	6
P26927	Hepatocyte growth factor-like protein	80.27	16	3	5
P27169	Serum paraoxonase/arylesterase 1	39.72	221	151	135
P27918	Properdin	51.24	14	22	21
P28072	Proteasome subunit beta type-6	25.34			3
P28328	Peroxisome biogenesis factor 2	34.82			2
P28332	Alcohol dehydrogenase 6	39.06			3
P29374	AT-rich interactive domain-containing protein 4A	142.66	6		
P29622	Kallistatin	48.51	78	50	38
P30043	Flavin reductase	22.11	3	6	
P30203	T-cell differentiation antigen CD6	71.76			2
P30273	High affinity immunoglobulin epsilon receptor subunit gamma	9.66			2
P30566	Adenylosuccinate lyase	54.85	2		
P30622	CAP-Gly domain-containing linker protein 1	162.15			2
P32119	Peroxiredoxin-2	21.88	9	3	8
P32942	Intercellular adhesion molecule 3	59.50	4		
P33151	Cadherin-5	87.46	2		
P33261	Cytochrome P450 2C19	55.89	3		
P33908	Mannosyl-oligosaccharide 1,2-alpha-mannosidase IA	72.92	17	9	
P34096	Ribonuclease 4	16.83	4		
P34925	Tyrosine-protein kinase RYK	67.46		14	13
P35318	ADM	20.41	2		
P35527	Keratin, type I cytoskeletal 9	62.03	305	101	93
P35542	Serum amyloid A-4 protein	14.74	47	80	62

P35579	Myosin-9	226.39		7	
P35858	Insulin-like growth factor-binding protein complex acid labile subunit	65.99	156	111	71
P35900	Keratin, type I cytoskeletal 20	48.46	6		
P35908	Keratin, type II cytoskeletal 2 epidermal	65.39	156	107	117
P36222	Chitinase-3-like protein 1	42.60	8	3	5
P36955	Pigment epithelium-derived factor	46.31	178	155	158
P36980	Complement factor H-related protein 2	30.63	84	82	97
P37802	Transgelin-2	22.38	13	4	3
P39060	Collagen alpha-1(XVIII) chain	178.08	2		
P41222	Prostaglandin-H2 D-isomerase	21.02	12		
P42261	Glutamate receptor 1	101.44		3	
P42330	Aldo-keto reductase family 1 member C3	36.82			3
P43121	Cell surface glycoprotein MUC18	71.56	5		
P43251	Biotinidase	61.09	11	11	3
P43652	Afamin	69.02	372	238	189
P46087	Putative ribosomal RNA methyltransferase NOP2	89.25			3
P47712	Cytosolic phospholipase A2	85.16	2		
P48454	Serine/threonine-protein phosphatase 2B catalytic subunit gamma isoform	58.09	2		
P48681	Nestin	177.33	2	2	2
P48740	Mannan-binding lectin serine protease 1	79.20	34	15	4
P49221	Protein-glutamine gamma-glutamyltransferase 4	77.10		3	
P49368	T-complex protein 1 subunit gamma	60.50	2		
P49454	Centromere protein F	367.54			3
P49711	Transcriptional repressor CTCF	82.73			2
P49747	Cartilage oligomeric matrix protein	82.81	3	10	2
P49798	Regulator of G-protein signaling 4	23.24		2	3
P49842	Serine/threonine-protein kinase 19	40.89		3	
P49908	Selenoprotein P	43.16	11	6	6
P51114	Fragile X mental retardation syndrome-related protein 1	69.68		2	3
P51587	Breast cancer type 2 susceptibility protein	383.99		2	3
P51608	Methyl-CpG-binding protein 2	52.41	2		4
P51841	Retinal guanylyl cyclase 2	124.74	4		
P51884	Lumican	38.40	82	61	58
P51970	NADH dehydrogenase [ubiquinone] 1 alpha subcomplex subunit 8	20.09		3	
P52747	Zinc finger protein 143	68.85	4		2
P52789	Hexokinase-2	102.31		2	
P53618	Coatomer subunit beta	107.07		2	
P54652	Heat shock-related 70 kDa protein 2	69.98	7		
P54868	Hydroxymethylglutaryl-CoA synthase, mitochondrial	56.60			2
P54886	Delta-1-pyrroline-5-carboxylate synthase	87.25	2		
P55056	Apolipoprotein C-IV	14.54	14	12	9
P55058	Phospholipid transfer protein	54.70	11	14	9
P55103	Inhibin beta C chain	38.21	6		6
P55290	Cadherin-13	78.24	2		3

P56703	Proto-oncogene Wnt-3	39.62			3
P57071	PR domain zinc finger protein 15	169.16		2	
P57771	Regulator of G-protein signaling 8	20.90	2	2	3
P58012	Forkhead box protein L2	38.75		2	
P60174	Triosephosphate isomerase	26.65	12	7	
P60709	Actin, cytoplasmic 1	41.71	126	85	59
P61088	Ubiquitin-conjugating enzyme E2 N	17.13	16	15	10
P61626	Lysozyme C	16.53	10	6	9
P61769	Beta-2-microglobulin	13.71	8	29	21
P62690	HERV-K_22q11.23 provirus ancestral Gag polyprotein	69.05			3
P62701	40S ribosomal protein S4, X isoform	29.58		2	
P62917	60S ribosomal protein L8	28.01	2	3	
P62937	Peptidyl-prolyl cis-trans isomerase A	18.00	9		
P63104	14-3-3 protein zeta/delta	27.73	6		5
P63267	Actin, gamma-enteric smooth muscle	41.85		35	
P67936	Tropomyosin alpha-4 chain	28.50	6	4	3
P68032	Actin, alpha cardiac muscle 1	41.99	76	24	56
P68366	Tubulin alpha-4A chain	49.89	2		
P69905	Hemoglobin subunit alpha	15.25	5		
P78356	Phosphatidylinositol-5-phosphate 4-kinase type-2 beta	47.35	2		3
P78417	Glutathione S-transferase omega-1	27.55	3		
P80108	Phosphatidylinositol-glycan-specific phospholipase D	92.28	59	57	51
P80188	Neutrophil gelatinase-associated lipocalin	22.57	6	3	4
P80723	Brain acid soluble protein 1	22.68		3	
P81605	Dermcidin	11.28	2	2	
P82094	TATA element modulatory factor	122.77		2	3
P85298	Rho GTPase-activating protein 8	53.45	2		
P98160	Basement membrane-specific heparan sulfate proteoglycan core protein	468.50		7	9
Q00796	Sorbitol dehydrogenase	38.30		2	
Q01850	Cerebellar degeneration-related protein 2	51.82		2	
Q02080	Myocyte-specific enhancer factor 2B	38.61		4	
Q02224	Centromere-associated protein E	316.22	2		
Q02985	Complement factor H-related protein 3	37.30	53	36	31
Q03591	Complement factor H-related protein 1	37.63	202	161	168
Q04756	Hepatocyte growth factor activator	70.64	35	19	15
Q06033	Inter-alpha-trypsin inhibitor heavy chain H3	99.79	153	110	105
Q06278	Aldehyde oxidase	147.82		2	2
Q07325	C-X-C motif chemokine 9	14.01		3	3
Q08357	Sodium-dependent phosphate transporter 2	70.3			2
Q08380	Galectin-3-binding protein	65.29	3	2	2
Q0VDD8	Dynein heavy chain 14, axonemal	399.64	3		
Q12815	Tastin	83.80		2	
Q12874	Splicing factor 3A subunit 3	58.81	8		2
Q12913	Receptor-type tyrosine-protein phosphatase eta	145.85	3	4	3
Q13023	A-kinase anchor protein 6	256.6	12		
Q13114	TNF receptor-associated factor 3	64.45	3		

Q13163	Dual specificity mitogen-activated protein kinase kinase 5	50.08		2	
Q13367	AP-3 complex subunit beta-2	118.98			2
Q13393	Phospholipase D1	124.11	4		
Q13439	Golgin subfamily A member 4	260.98	3		
Q13546	Receptor-interacting serine/threonine-protein kinase 1	75.88	2	2	
Q13790	Apolipoprotein F	33.44	18	10	4
Q13813	Spectrin alpha chain, brain	284.36			2
Q13822	Ectonucleotide pyrophosphatase/phospho diesterase family member 2	98.93	4	2	
Q13838	Spliceosome RNA helicase BAT1	48.96		5	
Q14126	Desmoglein-2	122.22		2	
Q14258	E3 ubiquitin/ISG15 ligase TRIM25	70.94			4
Q14451	Growth factor receptor-bound protein 7	59.64	2	2	3
Q14508	WAP four-disulfide core domain protein 2	12.98	2	6	4
Q14515	SPARC-like protein 1	75.16	3	2	
Q14520	Hyaluronan-binding protein 2	62.63	35	37	30
Q14576	ELAV-like protein 3	39.52		2	
Q14624	Inter-alpha-trypsin inhibitor heavy chain H4	103.29	650	631	466
Q14687	Genetic suppressor element 1	136.08	6	2	18
Q14690	Protein RRP5 homolog	208.57		3	
Q14766	Latent-transforming growth factor beta-binding protein 1	186.67		2	2
Q14919	Dr1-associated corepressor	22.34		2	
Q149M9	NACHT and WD repeat domain-containing protein 1	174.47		2	2
Q15113	Procollagen C-endopeptidase enhancer 1	47.94	4	7	
Q15139	Serine/threonine-protein kinase D1	101.64		2	
Q15349	Ribosomal protein S6 kinase alpha-2	83.19			2
Q15404	Ras suppressor protein 1	31.52	2		
Q15431	Synaptonemal complex protein 1	114.12	14	2	3
Q15582	Transforming growth factor-beta-induced protein ig-h3	74.63	21	17	18
Q15695	U2 small nuclear ribonucleoprotein auxiliary factor 35 kDa subunit-related protein 1	57.61	2		
Q15751	Probable E3 ubiquitin-protein ligase HERC1	531.89		2	
Q15797	Mothers against decapentaplegic homolog 1	52.23		2	6
Q15848	Adiponectin	26.40	8	2	
Q15878	Voltage-dependent R-type calcium channel subunit alpha-1E	261.56		2	
Q16134	Electron transfer flavoprotein-ubiquinone oxidoreductase, mitochondrial	68.45	3	2	2
Q16270	Insulin-like growth factor-binding protein 7	29.11		4	
Q16584	Mitogen-activated protein kinase kinase kinase 11	92.63	2	3	2
Q16610	Extracellular matrix protein 1	60.64	63	30	22
Q16635	Tafazzin	33.44			3
Q16666	Gamma-interferon-inducible protein 16	88.20		2	
Q16787	Laminin subunit alpha-3	366.41	2		4
Q2M2I5	Keratin, type I cytoskeletal 24	55.05	2		
Q2UY09	Collagen alpha-1(XXVIII) chain	116.58		2	

Q32NC0	UPF0711 protein C18orf21	24.81	4	3	7
Q3MIS6	Zinc finger protein 528	72.09	2		
Q3MJ40	Coiled-coil domain-containing protein 144B	82.90			2
Q3ZCT1	Zinc finger protein 260	47.19			22
Q4KMG0	Cell adhesion molecule-related/down-regulated by oncogenes	139.15	2		
Q4KWH8	1-phosphatidylinositol-4,5-bisphosphate phosphodiesterase eta-1	189.10	2		
Q53SF7	Cordon-bleu protein-like 1	131.71		2	
Q562R1	Beta-actin-like protein 2	41.98		14	
Q587J7	Tudor domain-containing protein 12	132.49		4	
Q5CZC0	Fibrous sheath-interacting protein 2	369.63		2	
Q5HYK3	Ubiquinone biosynthesis methyltransferase COQ5, mitochondrial	37.12		2	
Q5JRA6	Melanoma inhibitory activity protein 3	213.57	3		
Q5JS54	Proteasome assembly chaperone 4	13.77		6	2
Q5RHP9	Uncharacterized protein C1orf173	168.36		2	2
Q5SQS8	Uncharacterized protein C10orf120	39.24		2	
Q5SSJ5	Heterochromatin protein 1-binding protein 3	61.17	2		
Q5ST30	Valyl-tRNA synthetase, mitochondrial	118.41	2		
Q5SYB0	FERM and PDZ domain-containing protein 1	173.33	3		
Q5SZK8	FRAS1-related extracellular matrix protein 2	350.94	2		
Q5T0U0	Coiled-coil domain-containing protein 122	32.19	3		2
Q5T1M5	FK506-binding protein 15	133.55		2	
Q5T5Y3	Calmodulin-regulated spectrin-associated protein 1	177.86		2	2
Q5T655	Coiled-coil domain-containing protein 147	103.35	2		
Q5T6S3	PHD finger protein 19	65.55		2	
Q5T749	Keratinocyte proline-rich protein	64.09	4		
Q5T9S5	Coiled-coil domain-containing protein 18	168.86	4	4	
Q5TB80	Protein QN1 homolog	161.84			2
Q5TCX8	Mitogen-activated protein kinase kinase kinase	113.89		2	
Q5THR3	EF-hand calcium-binding domain-containing protein 6	172.82		4	
Q5VT28	Protein FAM27A/B/C	7.38		2	
Q5VTT2	Uncharacterized protein C9orf135	26.43		2	
Q5VTT5	Myomesin-3	162.09		2	
Q5VZY2	Phosphatidate phosphatase PPAPDC1A	30.37		2	
Q5XPI4	E3 ubiquitin-protein ligase RNF123	148.42	2		
Q63HK5	Teashirt homolog 3	118.49	2	3	3
Q659A1	NMDA receptor-regulated protein 2	109.94	2	2	
Q68BL8	Olfactomedin-like protein 2B	83.95	2	4	3
Q68CZ1	Protein fantom	151.11		10	6
Q68CZ6	HAUS augmin-like complex subunit 3	69.61		2	
Q6DT37	Serine/threonine-protein kinase MRCK gamma	172.35		4	
Q6EMK4	Vasorin	71.67	16		
Q6NZI2	Polymerase I and transcript release factor	43.45	2		
Q6P0Q8	Microtubule-associated serine/threonine-protein kinase 2	196.31	4		
Q6P1N0	Coiled-coil and C2 domain-containing protein 1A	104.00	2	2	2

Q6P2H8	Transmembrane protein 53	31.61		2	
Q6P2M8	Calcium/calmodulin-dependent protein kinase type 1B	38.48		2	
Q6P4R8	Nuclear factor related to kappa-B-binding protein	138.92		2	
Q6P6C2	Alkylated DNA repair protein alkB homolog 5	51.38		2	
Q6R327	Rapamycin-insensitive companion of mTOR	192.10	8	2	4
Q6RI45	Bromodomain and WD repeat-containing protein 3	203.50	16	8	
Q6TDU7	Cancer susceptibility candidate protein 1	83.17		3	
Q6UX06	Olfactomedin-4	57.24		2	
Q6UXB8	Peptidase inhibitor 16	49.44	7	6	2
Q6UXM1	Leucine-rich repeats and immunoglobulin-like domains protein 3	123.36		2	
Q6XZF7	Dynamin-binding protein	177.24	2		
Q6Y288	Beta-1,3-glucosyltransferase	56.53		2	
Q6ZN30	Zinc finger protein basonuclin-2	122.25	4	4	6
Q6ZQQ6	WD repeat-containing protein 87	332.97	2		
Q6ZR08	Dynein heavy chain 12, axonemal	356.71		2	
Q6ZS72	Putative uncharacterized protein C19orf35	50.48		3	
Q6ZVF9	G protein-regulated inducer of neurite outgrowth 3	82.39			2
Q6ZVN9	Putative uncharacterized protein FLJ42277	29.57		4	6
Q711Q0	Uncharacterized protein C10orf71	156.38	2		
Q71H61	Immunoglobulin-like domain-containing receptor 2	71.15		2	
Q76LX8	A disintegrin and metalloproteinase with thrombospondin motifs 13	153.50	2	2	
Q7L7X3	Serine/threonine-protein kinase TAO1	116.00		2	
Q7RTS6	Otopetrin-2	62.19	2		
Q7Z3J2	UPF0505 protein C16orf62	109.49	3	2	2
Q7Z3U7	Protein MON2 homolog	190.37		2	
Q7Z5A4	Putative testis serine protease 2	31.99		2	
Q7Z6I6	Rho GTPase-activating protein 30	118.52		3	
Q7Z6K3	Protein prenyltransferase alpha subunit repeat-containing protein 1	46.38	4	5	3
Q7Z7G0	Target of Nesh-SH3	118.57	2		
Q86UD1	Out at first protein homolog	30.67	4	2	2
Q86UE4	Protein LYRIC	63.80			3
Q86UT6	NLR family member X1	107.55	4	2	
Q86UV6	Tripartite motif-containing protein 74	28.53	6	20	14
Q86UX7	Fermitin family homolog 3	75.91	5		
Q86V48	Leucine zipper protein 1	120.20	3		
Q86VB7	Scavenger receptor cysteine-rich type 1 protein M130	125.36	20		
Q86VN1	Vacuolar protein-sorting-associated protein 36	43.79		2	
Q86WR0	Coiled-coil domain-containing protein 25	24.46		3	3
Q86XE5	Dihydrodipicolinate synthase-like, mitochondrial	35.23		2	
Q86YR7	Probable guanine nucleotide exchange factor MCF2L2	126.88		8	5
Q8IUB2	WAP four-disulfide core domain protein 3	24.67		2	
Q8IVF4	Dynein heavy chain 10, axonemal	514.51		2	
Q8IWJ2	GRIP and coiled-coil domain-containing protein 2	184.54	3	4	
Q8IWQ3	BR serine/threonine-protein kinase 2	81.58		2	

Q8IX04	Ubiquitin-conjugating enzyme E2 variant 3	52.23		7	5
Q8IX30	Signal peptide, CUB and EGF-like domain-containing protein 3	109.21	2		
Q8IXR9	Uncharacterized protein C12orf56	71.29	8	2	4
Q8IXS2	Coiled-coil domain-containing protein 65	57.26	3		
Q8IY21	Probable ATP-dependent RNA helicase DDX60	197.73	11	3	
Q8IY45	Protein AMN1 homolog	28.39			2
Q8IY51	Tigger transposable element-derived protein 4	57.43			2
Q8IY81	Putative rRNA methyltransferase 3	96.50		6	
Q8N157	Jouberin	137.03		3	
Q8N3K9	Cardiomyopathy-associated protein 5	448.94		2	
Q8N3L3	Beta-taxilin	76.47			3
Q8N4C9	Uncharacterized protein C17orf78	30.54		3	
Q8N584	Tetratricopeptide repeat protein 39C	65.83	2		
Q8N6C8	Leukocyte immunoglobulin-like receptor subfamily A member 3	47.44	8	2	
Q8N6R0	Methyltransferase-like protein 13	78.72	5		
Q8N895	Zinc finger protein 366	85.05		2	
Q8NA54	IQ and ubiquitin-like domain-containing protein	92.52		2	
Q8NB25	Protein FAM184A	132.88		3	5
Q8NBE8	Kelch-like protein 23	63.88		2	
Q8NBJ4	Golgi membrane protein 1	45.31	3	4	
Q8NCM2	Potassium voltage-gated channel subfamily H member 5	111.84	2		
Q8ND71	GTPase IMAP family member 8	74.84			2
Q8NDV7	Trinucleotide repeat-containing gene 6A protein	210.17	5	7	5
Q8NDW8	Tetratricopeptide repeat protein 21A	150.85			2
Q8NER1	Transient receptor potential cation channel subfamily V member 1	94.90		4	
Q8NHQ8	Ras association domain-containing protein 8	48.30		2	
Q8NHV4	Protein NEDD1	71.92	3		
Q8TAE8	Growth arrest and DNA damage-inducible proteins-interacting protein 1	25.37	6	3	2
Q8TC20	Cancer-associated gene 1 protein	90.19			2
Q8TD84	Down syndrome cell adhesion molecule-like protein 1	224.32		2	
Q8TDM6	Disks large homolog 5	213.74	4	3	3
Q8TDQ1	CMRF35-like molecule 1	32.31	3	5	
Q8TF21	Ankyrin repeat domain-containing protein 24	124.11	3	2	
Q8TF46	DIS3-like exonuclease 1	120.71			3
Q8WTR8	Netrin-5	53.14	3		
Q8WVX9	Fatty acyl-CoA reductase 1	59.32	2		2
Q8WX94	NACHT, LRR and PYD domains-containing protein 7	111.73		3	
Q8WYP3	Ras and Rab interactor 2	100.10	2		
Q8WZ42	Titin	3813.81	4		
Q92466	DNA damage-binding protein 2	47.83	3		4
Q92556	Engulfment and cell motility protein 1	83.78		3	
Q92615	La-related protein 4B	80.50	2		
Q92625	Ankyrin repeat and SAM domain-containing protein 1A	123.03	3	2	

Q92670	Putative zinc finger protein 75C	49.72			2
Q92736	Ryanodine receptor 2	564.38		2	2
Q92794	Histone acetyltransferase MYST3	224.89		3	
Q92817	Envoplakin	231.46		2	2
Q92820	Gamma-glutamyl hydrolase	35.94		2	
Q92889	DNA repair endonuclease XPF	104.42		3	
Q92954	Proteoglycan 4	150.98	40	18	12
Q93084	Sarcoplasmic/endoplasmic reticulum calcium ATPase 3	113.90		2	
Q96AV8	Transcription factor E2F7	99.78	2		
Q96BP3	Peptidylprolyl isomerase domain and WD repeat-containing protein 1	73.53	2		
Q96BT7	Alkylated DNA repair protein alkB homolog 8	75.16	2	2	
Q96BY6	Dedicator of cytokinesis protein 10	249.15			7
Q96CN5	Leucine-rich repeat-containing protein 45	75.90	2		
Q96CV9	Optineurin	65.88	5	8	8
Q96E66	Leucine-rich repeat-containing protein 51	22.19		2	
Q96EA4	Protein Spindly	70.13	2		
Q96FZ7	Charged multivesicular body protein 6	23.47	4	8	2
Q96I24	Far upstream element-binding protein 3	61.60			4
Q96IY4	Carboxypeptidase B2	48.38	18	4	4
Q96JH7	Deubiquitinating protein VCIP135	134.24		2	
Q96JI7	Spatacsin	278.69	8	2	
Q96KK5	Histone H2A type 1-H	13.90		5	4
Q96KN2	Beta-Ala-His dipeptidase	56.66	40	49	29
Q96KQ7	Histone-lysine N-methyltransferase, H3 lysine-9 specific 3	132.29	2		
Q96KR1	Zinc finger RNA-binding protein	116.94		3	2
Q96KR7	Phosphatase and actin regulator 3	62.51	2		
Q96LW2	Uncharacterized serine/threonine-protein kinase SgK494	31.02			3
Q96MN2	NACHT, LRR and PYD domains-containing protein 4	113.34		2	
Q96N67	Dedicator of cytokinesis protein 7	242.41	2		
Q96P70	Importin-9	115.89		2	
Q96PD5	N-acetylmuramoyl-L-alanine amidase	62.18	100	78	66
Q96PE3	Type I inositol-3,4-bisphosphate 4-phosphatase	109.89		8	
Q96PN6	Adenylate cyclase type 10	187.01		2	
Q96PU8	Protein quaking	37.65			2
Q96PZ7	CUB and sushi domain-containing protein 1	388.62	3		5
Q96Q04	Serine/threonine-protein kinase LMTK3	153.57	2	2	
Q96RW7	Hemicentin-1	613.00	3	3	3
Q96T60	Bifunctional polynucleotide phosphatase/kinase	57.04		6	
Q99708	Retinoblastoma-binding protein 8	101.88			2
Q99784	Noelin	55.31	9		
Q99969	Retinoic acid receptor responder protein 2	18.61		6	
Q99996	A-kinase anchor protein 9	453.39		2	
Q9BQ16	Testican-3	49.40	3		
Q9BQ52	Zinc phosphodiesterase ELAC protein 2	92.16	2	3	2
Q9BQA9	Uncharacterized protein C17orf62	20.76	3		

Q9BRP8	Partner of Y14 and mago	22.64			2
Q9BRQ6	Coiled-coil-helix-coiled-coil-helix domain-containing protein 6	26.44	3		
Q9BSJ5	Uncharacterized protein C17orf80	67.25			2
Q9BSK4	Protein fem-1 homolog A	73.59			3
Q9BU64	Centromere protein O	33.76			2
Q9BW62	Katanin p60 ATPase-containing subunit A-like 1	55.36		4	4
Q9BWG6	Sodium channel modifier 1	25.93		2	
Q9BXR6	Complement factor H-related protein 5	64.38	47	21	37
Q9BXT5	Testis-expressed sequence 15 protein	315.16	2		
Q9BYC8	39S ribosomal protein L32, mitochondrial	21.39			2
Q9BYW2	Histone-lysine N-methyltransferase SETD2	287.42	2	4	
Q9BYX2	TBC1 domain family member 2A	104.04		2	2
Q9BYX4	Interferon-induced helicase C domain-containing protein 1	116.61			2
Q9BZR9	Tripartite motif-containing protein 8	61.45			2
Q9C093	Sperm flagellar protein 2	209.68		5	
Q9C0B5	Probable palmitoyltransferase ZDHHC5	77.50	2		
Q9C0J8	WD repeat-containing protein 33	145.80			2
Q9H0C1	Zinc finger MYND domain-containing protein 12	41.76		2	
Q9H1M0	Nucleoporin-62 C-terminal-like protein	20.81		2	
Q9H254	Spectrin beta chain, brain 3	288.81	3		
Q9H2P0	Activity-dependent neuroprotector homeobox protein	123.49		8	
Q9H2U9	Disintegrin and metalloproteinase domain-containing protein 7	85.61			2
Q9H3S4	Thiamin pyrophosphokinase 1	27.25			2
Q9H4A9	Dipeptidase 2	53.27		2	
Q9H4B4	Serine/threonine-protein kinase PLK3	71.58	2		
Q9H4G4	Golgi-associated plant pathogenesis-related protein 1	17.21	6		
Q9H6S1	5-azacytidine-induced protein 2	44.91	2		
Q9H7P6	Multivesicular body subunit 12B	35.60		6	
Q9H7U1	Protein FAM190B	93.49	2		
Q9H814	Phosphorylated adapter RNA export protein	44.38		4	4
Q9HBH9	MAP kinase-interacting serine/threonine-protein kinase 2	51.84		2	
Q9HBM0	Vezatin	88.61			2
Q9HC10	Otoferlin	226.61		2	
Q9HCH5	Synaptotagmin-like protein 2	104.87	2		
Q9HCM4	Band 4.1-like protein 5	81.80		4	2
Q9HDC9	Adipocyte plasma membrane-associated protein	46.45	3	2	
Q9NP78	ATP-binding cassette sub-family B member 9	84.42	5	4	3
Q9NPC2	Potassium channel subfamily K member 9	42.24			2
Q9NQC1	Protein Jade-2	87.41			2
Q9NQC8	UPF0360 protein C11orf60	34.26	2	2	
Q9NR11	Zinc finger protein 302	54.78	2	2	4
Q9NUY8	TBC1 domain family member 23	78.27			2
Q9NWA0	Mediator of RNA polymerase II transcription subunit 9	16.39		5	
Q9NWT1	p21-activated protein kinase-interacting protein 1	43.94		4	

Q9NX36	DnaJ homolog subfamily C member 28	45.78			2
Q9NXD2	Myotubularin-related protein 10	88.22	9	7	7
Q9NXS3	Kelch-like protein 28	64.15	14	6	8
Q9NY99	Gamma-2-syntrophin	60.18	2		
Q9NYL9	Tropomodulin-3	39.57	6		
Q9NZH7	Interleukin-1 family member 8	18.51		2	
Q9NZP8	Complement C1r subcomponent-like protein	53.46	30	33	36
Q9P0N9	TBC1 domain family member 7	33.95			2
Q9P1W3	Transmembrane protein 63C	93.26	2		
Q9P206	Uncharacterized protein KIAA1522	107.03	4		
Q9P219	Protein Daple	228.07	2		
Q9P225	Dynein heavy chain 2, axonemal	507.37	2		
Q9P278	Folliculin-interacting protein 2	122.04		2	
Q9P281	BAH and coiled-coil domain-containing protein 1	276.76	2	4	2
Q9P2E3	NFX1-type zinc finger-containing protein 1	220.08		2	
Q9P2G1	Ankyrin repeat and IBR domain-containing protein 1	121.92		3	
Q9P2G4	Uncharacterized protein KIAA1383	100.28	2		
Q9P2K5	Myelin expression factor 2	64.08	2		
Q9UBI1	COMM domain-containing protein 3	22.14	4		
Q9UBL0	cAMP-regulated phosphoprotein 21	89.14	2		
Q9UBT6	DNA polymerase kappa	98.75	12	12	5
Q9UDT6	CAP-Gly domain-containing linker protein 2	115.77		7	
Q9UDV7	Zinc finger protein 282	74.25	2		
Q9UGM5	Fetuin-B	42.03	32	15	16
Q9UHG3	Prenylcysteine oxidase 1	56.60	11	2	
Q9UHQ1	Nuclear prelamin A recognition factor	51.12	3		
Q9UIR0	Butyrophilin-like protein 2	50.4		2	
Q9UK55	Protein Z-dependent protease inhibitor	50.67	93	25	38
Q9UKJ3	G patch domain-containing protein 8	164.10		2	
Q9UKN7	Myosin-XV	395.04			3
Q9ULC5	Long-chain-fatty-acid--CoA ligase 5	75.94			2
Q9ULJ1	Outer dense fiber protein 2-like	73.68	3	5	
Q9ULP9	TBC1 domain family member 24	62.88			2
Q9ULT8	E3 ubiquitin-protein ligase HECTD1	289.41	3	3	2
Q9UM54	Myosin-VI	149.60	3		
Q9UM63	Zinc finger protein PLAGL1	50.79			5
Q9UMQ3	Homeobox protein BarH-like 2	31.17		2	
Q9UNW1	Multiple inositol polyphosphate phosphatase 1	55.02	3		
Q9UPA5	Protein bassoon	416.21	3		
Q9UPS8	Ankyrin repeat domain-containing protein 26	196.20	2		4
Q9UPY8	Microtubule-associated protein RP/EB family member 3	31.96		2	
Q9UPZ3	Hermansky-Pudlak syndrome 5 protein	127.37	3		
Q9UPZ6	Thrombospondin type-1 domain-containing protein 7A	185.24	5		
Q9Y247	Protein FAM50B	38.68	2		
Q9Y265	RuvB-like 1	50.20		4	4
Q9Y274	Type 2 lactosamine alpha-2,3-sialyltransferase	38.19	3		
Q9Y279	V-set and immunoglobulin domain-containing protein 4	43.96		6	

Q9Y2L5	Protein TRS85 homolog	160.90	2		
Q9Y2L6	FERM domain-containing protein 4B	111.56	3	5	4
Q9Y2V0	Uncharacterized protein C15orf41	32.24			3
Q9Y3B7	39S ribosomal protein L11, mitochondrial	20.67	5		
Q9Y3L5	Ras-related protein Rap-2c	20.73	5		
Q9Y483	Metal-response element-binding transcription factor 2	67.05		2	
Q9Y490	Talin-1	269.60	2	2	
Q9Y4B4	Helicase ARIP4	162.67	8		4
Q9Y4B5	Uncharacterized protein KIAA0802	208.65		5	
Q9Y4C1	Lysine-specific demethylase 3A	147.23	5		3
Q9Y4E6	WD repeat-containing protein 7	163.71	3		
Q9Y5G5	Protocadherin gamma-A8	101.42		4	
Q9Y5Y7	Lymphatic vessel endothelial hyaluronic acid receptor 1	35.19	18	5	
Q9Y616	Interleukin-1 receptor-associated kinase 3	67.71	3		
Q9Y623	Myosin-4	222.93			2
Q9Y6D9	Mitotic spindle assembly checkpoint protein MAD1	83.02		2	
Q9Y6F1	Poly [ADP-ribose] polymerase 3	60.03	7	2	
Q9Y6M1	Insulin-like growth factor 2 mRNA-binding protein 2	66.08	4		2
Q9Y6R7	IgGFc-binding protein	571.64	15	6	
Q9Y6V0	Protein piccolo	566.31	8		
Q9Y6W5	Wiskott-Aldrich syndrome protein family member 2	54.25	2		

[a] Accession number from the Swiss-Prot database (human),

[b] Molecular weight,

[c] Spectral counts for each identified proteins in each workflow replicate,

[d] Workflow replicate.
