# Supplementary material for: Additions to the Human Plasma Proteome via a Tandem MARS Depletion iTRAQ-Based Workflow
Source: Int J Proteomics. 2013 Feb 19;2013:654356. doi: 10.1155/2013/654356 (PMC3590782; doi:10.1155/2013/654356)
Supplement: Supplementary file 1 — “The Supplementary Material provides the following information: Table S1) Depletion efficiency of the six high abundance proteins with MD and TMD; Table S2) A list of proteins identified with corresponding spectral counts in each workflow replicate; Table S3) CV values for proteins quantified in workflow replicates; Table S4) CV values for proteins quantified in at least two technical replicates, and Figure S1) The distribution of SD values for proteins quantified in all workflow replicates as a function of log2 transformed ratios.” [file 654356.f1.zip › TableS3.pdf]

Table S3. CV values obtained using different reference channels

	**CV (all 3 WR)[a]**		**CV (any 2 WR)[b]**		**CV (at least 2 WR)[c]**	
	**mean**	**median**	**mean**	**median**	**mean**	**median**
$\mathbf{I_{115}/I_{114}}$	0.16	0.13	0.30	0.23	0.21	0.16
$\mathbf{I_{116}/I_{114}}$	0.13	0.11	0.20	0.15	0.15	0.12
$\mathbf{I_{117}/I_{114}}$	0.11	0.09	0.18	0.15	0.13	0.10
$\mathbf{I_{114}/I_{115}}$	0.17	0.14	0.31	0.21	0.22	0.16
$\mathbf{I_{116}/I_{115}}$	0.15	0.12	0.26	0.18	0.19	0.14
$\mathbf{I_{117}/I_{115}}$	0.17	0.14	0.26	0.18	0.21	0.16
$\mathbf{I_{114}/I_{116}}$	0.13	0.10	0.20	0.15	0.15	0.11
$\mathbf{I_{115}/I_{116}}$	0.16	0.12	0.26	0.18	0.19	0.14
$\mathbf{I_{117}/I_{116}}$	0.13	0.11	0.20	0.15	0.15	0.12
$\mathbf{I_{114}/I_{117}}$	0.11	0.09	0.18	0.15	0.13	0.10
$\mathbf{I_{115}/I_{117}}$	0.17	0.14	0.28	0.22	0.21	0.16
$\mathbf{I_{116}/I_{117}}$	0.13	0.12	0.20	0.13	0.15	0.12

[a]CV, values obtained for proteins quantified in all three workflow replicates (N=139),
[b]CV, values obtained for proteins quantified in any two workflow replicates (N=71),
[c]CV, values obtained for proteins quantified in at least 2 workflow replicates (N=210).
