# Supplementary material for: Additions to the Human Plasma Proteome via a Tandem MARS Depletion iTRAQ-Based Workflow
Source: Int J Proteomics. 2013 Feb 19;2013:654356. doi: 10.1155/2013/654356 (PMC3590782; doi:10.1155/2013/654356)
Supplement: Supplementary file 1 — “The Supplementary Material provides the following information: Table S1) Depletion efficiency of the six high abundance proteins with MD and TMD; Table S2) A list of proteins identified with corresponding spectral counts in each workflow replicate; Table S3) CV values for proteins quantified in workflow replicates; Table S4) CV values for proteins quantified in at least two technical replicates, and Figure S1) The distribution of SD values for proteins quantified in all workflow replicates as a function of log2 transformed ratios.” [file 654356.f1.zip › TableS4.pdf]

Table S4. CV values for technical replicates using ion 114 as reference channel.

	CV (WR1)[a]		CV (WR2)[b]		CV (WR3)[c]	
	**mean**	**median**	**mean**	**median**	**mean**	**median**
I_{115}/I_{114}	0.10	0.08	0.10	0.09	0.10	0.08
I_{116}/I_{114}	0.09	0.08	0.09	0.08	0.09	0.07
I_{117}/I_{114}	0.08	0.07	0.10	0.08	0.11	0.09

[a]CV, values obtained for proteins quantified in at least two technical replicates in workflow replicate 1 (N=187),

[b]CV, values obtained for proteins quantified in at least two technical replicates in workflow replicate 2 (N=153),

[c]CV, values obtained for proteins quantified in at least two technical replicates in workflow replicate 3 (N=153).
