# Supplementary figures and images for: Additions to the Human Plasma Proteome via a Tandem MARS Depletion iTRAQ-Based Workflow
Source: Int J Proteomics. 2013 Feb 19;2013:654356. doi: 10.1155/2013/654356 (PMC3590782; doi:10.1155/2013/654356)

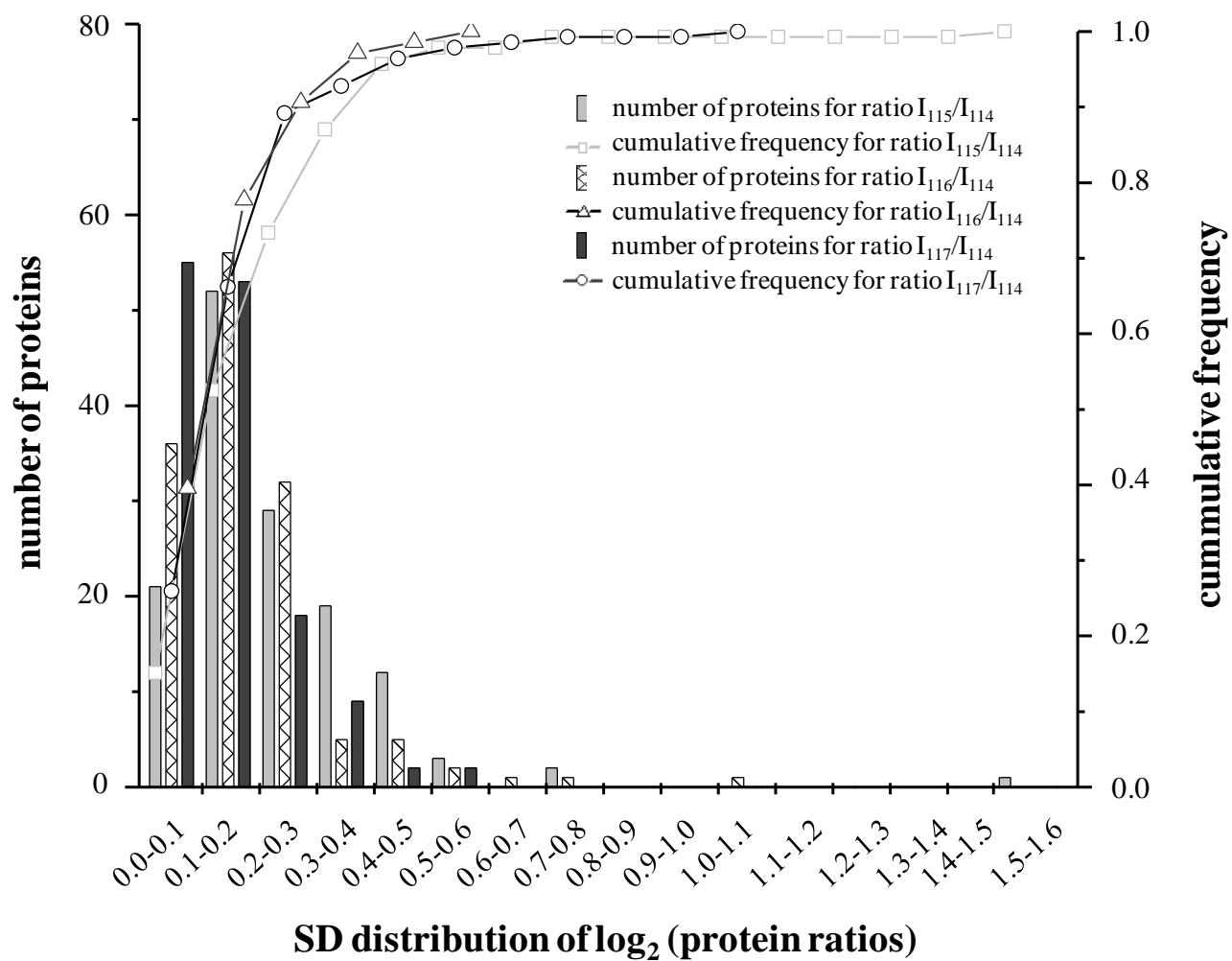

Supplement: Supplementary file 1 — “The Supplementary Material provides the following information: Table S1) Depletion efficiency of the six high abundance proteins with MD and TMD; Table S2) A list of proteins identified with corresponding spectral counts in each workflow replicate; Table S3) CV values for proteins quantified in workflow replicates; Table S4) CV values for proteins quantified in at least two technical replicates, and Figure S1) The distribution of SD values for proteins quantified in all workflow replicates as a function of log2 transformed ratios.” [file 654356.f1.zip › FigureS1.pdf]
